# BAP1 Tumour Predisposition Syndrome Due to Whole BAP1 Gene Deletion

**DOI:** 10.1155/2022/5503505

**Published:** 2022-09-13

**Authors:** Dinusha Pandithan, Sonja Klebe, Grace McKavanagh, Lesley Rawlings, Sui Yu, Jillian Nicholl, Nicola Poplawski

**Affiliations:** ^1^Adult Genetics Unit, Royal Adelaide Hospital, Adelaide, South Australia, Australia; ^2^Anatomical Pathology, SA Pathology and Flinders University, Flinders Medical Centre Site, Bedford Park, South Australia, Australia; ^3^Molecular Pathology, Mater Hospital Brisbane, South Brisbane, Australia; ^4^Genetics and Molecular Pathology, SA Pathology, Adelaide Site, South Australia, Australia; ^5^Adelaide Medical School, University of Adelaide, Adelaide, South Australia, Australia

## Abstract

BRCA-1-associated protein-1 (BAP1) tumour predisposition syndrome (BAP1-TPDS) is a dominant hereditary cancer syndrome. The full spectrum of associated malignancies is yet to be fully characterised. We detail the phenotypic features of the first reported family with a whole BAP1 gene deletion. This report also adds to the emerging evidence that the rhabdoid subtype of meningioma is a part of the clinical spectrum of this tumour predisposition syndrome.

## 1. Introduction

BRCA1-associated protein-1 (BAP1 OMIM #603089) tumour predisposition syndrome (BAP1-TPDS OMIM #614327) is an autosomal dominant hereditary cancer syndrome caused by heterozygous germline loss of function BAP1 pathogenic variants [1]. The BAP1 protein functions as a tumour suppressor and in heterozygous carrier tumour development follows somatic inactivation of the wild-type allele [2]. The most commonly associated tumour include uveal melanoma, malignant mesothelioma, cutaneous melanoma, clear cell renal cell carcinoma, and cutaneous basal cell carcinoma (BCC). Affected individuals are also susceptible to the development of cutaneous BAP1-inactivated melanocytic tumours (BIMT) that were previously described as atypical Spitz tumours, with a younger age of onset and significantly higher penetrance than a number of other BAP1-TPDS tumours [3, 4]. The complete tumour phenotype is yet to be fully characterised with recent reports suggesting the inclusion of rhabdoid meningioma, cholangiocarcinoma, breast cancer, thyroid cancer, neuroendocrine tumours, and nonsmall-cell lung adenocarcinoma [5–8].

Tumour BAP1 biallelic loss can be screened for with immunohistochemistry (IHC), looking for absent (abnormal) nuclear staining [2]. A range of somatic and germline BAP1 pathogenic variants have been described including large deletions of single or multiple exons causing loss of the N-terminal region, small insertions or deletions leading to frameshift and protein truncation, nonsense, missense, and splice site variants [9]. BAP1-TPDS-affected individuals inherit a germline loss-of-function BAP1 allele, with over 70% of BAP1 germline variants leading to truncation [10, 11]. The earliest reported age of tumour development is 15 years, and there is high disease penetrance with up to 80% of BAP1 mutation carriers developing at least one of the associated cancers [3, 9]. No clear genotype-phenotype correlation has emerged.

Here, we report a family with BAP1-TPDS due to a 151kb deletion at chromosome 3p21.1 involving the entire BAP1 gene; the proband was included in an international report of multiple BAP1-TPDS families [8], but a detailed description of the proband and family phenotype, and the molecular findings was not presented.

## 2. Clinical Report

**Figure 1:**
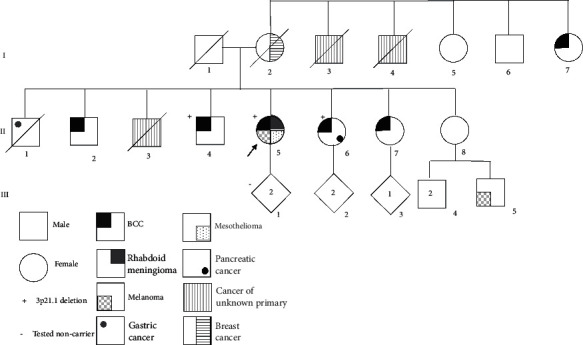
Details of her family cancer history and the results of available family segregation testing for the 3p21.1 deletion.

A 66-year-old female (proband II-5, Figure 1) presented with a personal history of left choroidal melanoma, cutaneous BCC, and multiple recurrent meningioma in the context of a family history of various cancer types (Figure 1). She was first diagnosed with sphenoid wing and temporal convexity meningiomas at the age of 56 (two separate tumours). These were recurrent in nature and she underwent multiple excisions with confirmation on histopathology of the WHO grade III rhabdoid meningioma in all surgical samples. She was clinically diagnosed with a left choroidal melanoma at the age of 58 which was treated with plaque radiotherapy, and therefore, no histopathological confirmation was available. At the age of 60, she had a nasal cutaneous BCC excised. BAP1 IHC was performed on meningioma and BCC tissue and demonstrated loss of nuclear staining. At the age of 68, she developed a right-sided pleural mass with associated pleural effusion. Histopathological examination of a pleural biopsy confirmed malignant mesothelioma of epithelioid type. At present, at the age of 71, she has advanced metastatic disease including chronic bilateral pleural effusions and is managed on carboplatin and pemetrexed. An echocardiogram at the age of 67 showed mild interventricular septal thickening (1.3 cm) of unknown aetiology (no hypertension).

**Figure 2:**
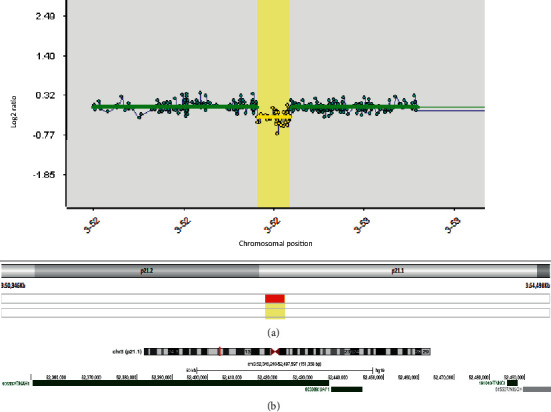
(a) An array profile of the deletion and (b) the breakpoints of the deletion and involved OMIM morbid genes.

BAP1 gene mutation analysis was arranged by next generation sequencing (NGS) and multiplex ligation-dependent probe amplification (MLPA) (Salsa MLPA kit P417-B1). Copy number variant (CNV) analysis by NGS was not available at the time of testing. Sequence analysis did not detect a pathogenic sequence variant. MLPA detected a germline heterozygous whole gene deletion, BAP1 (NM_004656.3): c.(?_-1)_(^*∗*^1_?) del. The SNP array (Illumina Infinium CytoSNP-850K BeadChip) defined a 151kb deletion of chromosome 3p21.1 (Figure 2(a)) involving six HGNC genes, including four OMIM genes, two of which are OMIM morbid genes (Figure 2(b)), BAP1 and *TNNC1* ((Human Feb 2009 (hg19) assembly; chr3:52346240-52497597). *TNNC1* (OMIM #191040) encodes for cardiac troponin C and is associated with sarcomeric cardiomyopathies (OMIM #611879, #613243), which can have an earlier age of disease onset and a more severe prognosis when compared to individuals with other affected troponin subunits, *TNNT2* or *TNNI3* [12]. Whole *TNNC1* gene deletions have not been reported to date. Using American College of Medical Genetics and Genomics (ACMG) criteria the deletion is classified as pathogenic (PVS1, PM2, PM4, and PP4).

Her heterozygous sister (Figure 1; II-6) died of hepatoid carcinoma of the pancreas at 59 years; the hepatoid carcinoma had loss of nuclear staining for BAP1 on IHC. Her heterozygous brother (II-4) had a cutaneous BCC at 59 years; this tumour had loss of nuclear labelling for BAP1 on IHC. An additional brother was diagnosed with and died from a metastatic carcinoma of undeterminable primary site; IHC performed the following detection of the familial BAP1 variant confirmed loss of BAP1 nuclear staining of tumour tissue; consent was not able to be obtained for molecular testing. In addition to breast cancer, her mother (I-2) had a history of enucleation of one eye for unconfirmed reasons. No DNA is available from her for molecular testing.

Furthermore, reported cancer history in untested family members includes a brother (II-2), a sister (II-7), and a maternal aunt (I-7) diagnosed with a cutaneous BCC at 59 years, 50 years, and unknown age ,respectively, and another brother (II-1) who died of gastric cancer at 48 years. A nephew (III-5) is reported to have had malignant melanoma at 22 years.

There was no other family history of note, including no family history of hypertrophic cardiomyopathy (HCM), although the family was not systematically screened with echocardiography. Incomplete penetrance and variable expression have been reported with *TNNC1* variants, which may explain the absence of a family history and the proband's mild cardiac phenotype [13].

## 3. Discussion

In 2018, Walpole et al collated the clinical and molecular phenotypes of 106 published and 75 unpublished BAP1 germline variant-positive families worldwide. In total, there were 104 unique null and 36 unique missense variants that the authors considered pathogenic or likely pathogenic using the modified ACMG criteria. We reported the detailed cancer and molecular phenotype of the only family included in that report with a whole BAP1 gene deletion. The cancer phenotype in this family included multiple rhabdoid meningioma in the proband.

The “core tumours” of BAP1-TPDS are uveal melanoma, malignant mesothelioma, cutaneous melanoma, and renal cell carcinoma. There is also evidence of increased risk of other tumour types, including meningioma [5, 6, 8].

The frequency of somatic BAP1 variants in all types of meningioma is <1% [14]. In the 104 null-variant BAP1 families described by Walpole et al., meningioma was documented in 8.5% of probands (*N* = 12), 2.2% of nonproband variant carriers (*N* = 4), and 1.3% of nongenotyped relatives (*N* = 7). BAP1 immunohistochemistry was reported for three of the 23 meningiomas, and expression was absent in all three. The histological subtype of the meningioma was not reported.

The World Health Organization classification of tumours designates rhabdoid meningioma as a high-grade tumour, despite only a subset being clinically aggressive with a high rate of postsurgical recurrence. Shankar et al. [15] demonstrated a correlation between the loss of the BAP1 expression in meningioma tumour and the extent of rhabdoid features. In addition, long term follow-up showed that in comparison to patients with BAP1-retained grade II and III meningiomas, BAP1-deficient meningiomas were more likely to recur and were clinically more aggressive [15]. Of relevance, inactivating BAP1 mutations or deletions paired with chromosome 3 loss of heterozygosity were identified in all 12 rhabdoid meningioma samples (5 patients) available for testing, indicating biallelic BAP1 “hits” are common in rhabdoid meningioma. Three of these 5 patients had constitutional DNA available for testing, one of whom was confirmed to have a germline BAP1 variant [15]. Subsequently, Shankar and Santagata [16] reported three additional patients with rhabdoid meningioma and germline BAP1 variants.

Ravanpay et al. [17] reported an adolescent patient (age 12 years) with rhabdoid meningioma and biallelic BAP1 variants, the truncating variant c.1174C > *T* and a chromosome 3p deletion that included BAP1. Only the truncating BAP1 variant was detected in germline DNA, indicating the 3p deletion was somatic.

Here, we report a woman with germline heterozygous BAP1 variant (a whole gene deletion) and multiple BAP1-deficient rhabdoid meningioma that showed aggressive behaviour. Our family adds to the emerging evidence that meningioma, in particular the high-grade rhabdoid subtype, is a part of the clinical spectrum of BAP1-TPDS. At present, due to the rare occurrence of BAP1 germline variants in general and BAP1-deficient rhabdoid meningioma specifically, the role of systematic neuroimaging for surveillance in BAP1-TPDS is unclear [18]. As more BAP1-TPDS families are identified and reported, evidence-based surveillance plans for this tumour predisposition syndrome will evolve.

Germline copy number variants (CNVs) are the genetic cause of multiple hereditary diseases [19]. Although next-generation sequencing (NGS) is the technology of choice for variant-identification in Mendelian disorders, there remain limitations in its ability to detect large rearrangements and large deletions and duplication. Although the tools for CNV detection using NGS platforms are improving, the gold standard for CNV detection are MLPA and array comparative genomic hybridisation (aCGH) [20]. This report confirms the need to include copy number variant (CNV) analysis in the molecular work-up of suspected BAP1-TPDS.

## 4. Consent

Written informed consent for publication of clinical information was obtained from each individual whose genetic results are included.

## Data Availability

The data that support the findings of this study are available from the corresponding author upon reasonable request.
